# Favorable response to tianeptine in an elderly patient: a case report

**DOI:** 10.1192/j.eurpsy.2026.11573

**Published:** 2026-07-31

**Authors:** M. Menendez Munoz, E. L. Bardón, Y. B. García, I. G. Rodríguez

**Affiliations:** Psychiatry, CAULE, León, Spain

## Abstract

**Introduction:**

A 74-year-old male with chronic anxiety and a previous brief depressive episode presents with persistent major depression, severe anxiety, and cognitive complaints including memory and attention difficulties worsened by benzodiazepine use. He reports insomnia, anhedonia, and difficulty managing daily activities. Despite treatment with high-dose SSRIs and benzodiazepines, symptoms and functional impairment persist. No clear psychosocial triggers are identified. Family reports sedation, confusion, and a recent fall linked to benzodiazepines. The patient remains independent in basic activities of daily living.

His medical history includes controlled hypertension, dyslipidemia, anticoagulated atrial fibrillation, and knee osteoarthritis. He denies substance use and has no known drug allergies or family psychiatric history. Comprehensive workup including labs, hormonal profile, urine toxicology, and brain CT is unremarkable.

**Objectives:**

To evaluate the discontinuation of benzodiazepines and the initiation of alternative pharmacological strategies with a more favorable safety and tolerability profile in the elderly population, such as tianeptine.

**Methods:**

The patient was alert, oriented, and cooperative, exhibiting sad, blunted affect and psychomotor retardation. Speech was coherent, monotone, and slowed. He demonstrated depressed, apathetic mood with anhedonia, hopelessness, and severe mixed anxiety. Attention and concentration were impaired; memory and orientation intact. No suicidal ideation. Insomnia, adequate oral intake, and social withdrawal were present. Intellectual function and judgment remained intact.

The clinical diagnosis was moderate depressive episode (ICD-10 F32.1) with severe anxiety and cognitive impairment secondary to benzodiazepine use; due to age and comorbidities, **tianeptine 12.5 mg twice daily** was initiated, benzodiazepines were gradually discontinued, the SSRI was stopped, a non-benzodiazepine hypnotic was prescribed nocturnally, and supportive psychotherapy with cognitive remediation was implemented.

**Results:**

After four weeks, the patient showed improved mood, less anxiety, better sleep, and enhanced attention and memory. These improvements continued at six weeks. The family reported reduced anxiety without side effects like drowsiness or confusion seen with previous benzodiazepines. By two and a half months, the patient resumed social activities such as golf and socializing, with no major side effects or cognitive decline. The patient continues both medication and psychological therapy.

**Conclusions:**

Tianeptine is an effective treatment for elderly patients with comorbid depression and anxiety, especially when benzodiazepines are poorly tolerated. It provides **strong anxiolytic effects without sedation or cognitive impairment**. Its glutamatergic action improves mood, anxiety, and cognition with minimal side effects. Tianeptine can be a first-line option in this population.

**Disclosure of Interest:**

None Declared

